# Identification of Salt Tolerance-Related *NAC* Genes in Wheat Roots Based on RNA-Seq and Association Analysis

**DOI:** 10.3390/plants14152318

**Published:** 2025-07-27

**Authors:** Lei Zhang, Aili Wei, Weiwei Wang, Xueqi Zhang, Zhiyong Zhao, Linyi Qiao

**Affiliations:** 1 College of Biological Sciences and Technology, Taiyuan Normal University, Taiyuan 030619, China; lei.zhang@tynu.edu.cn (L.Z.);; 2 Cangzhou Academy of Agriculture and Forestry Sciences, Cangzhou 061001, China; 3 Shanxi Key Laboratory of Crop Genetics and Molecular Improvement, College of Agronomy, Shanxi Agricultural University, Taiyuan 030031, China; 4 Institute of Cotton Research, Shanxi Agricultural University, Yuncheng 044000, China

**Keywords:** wheat, *NAC* genes, salt stress, transcriptome, association analysis

## Abstract

Excavating new salt tolerance genes and utilizing them to improve salt-tolerant wheat varieties is an effective way to utilize salinized soil. The *NAC* gene family plays an important role in plant response to salt stress. In this study, 446 *NAC* sequences were isolated from the whole genome of common wheat and classified into 118 members based on subgenome homology, named *TaNAC1* to *TaNAC118*. Transcriptome analysis of salt-tolerant wheat breeding line CH7034 roots revealed that 144 of the 446 *TaNAC* genes showed significant changes in expression levels at least two time points after NaCl treatment. These differentially expressed *TaNACs* were divided into four groups, and Group 4, containing the largest number of 78 genes, exhibited a successive upregulation trend after salt treatment. Single nucleotide polymorphisms (SNPs) of the *TaNAC* gene family in 114 wheat germplasms were retrieved from the public database and were subjected to further association analysis with the relative salt-injury rates (RSIRs) of six root phenotypes, and then 20 SNPs distributed on chromosomes 1B, 2B, 2D, 3B, 3D, 5B, 5D, and 7A were correlated with phenotypes involving salt tolerance (*p* < 0.0001). Combining the results of RT-qPCR and association analysis, we further selected three *NAC* genes from Group 4 as candidate genes that related to salt tolerance, including *TaNAC26-D3.2*, *TaNAC33-B*, and *TaNAC40-B*. Compared with the wild type, the roots of the *tanac26-d3.2* mutant showed shorter length, less volume, and reduced biomass after being subjected to salt stress. Four SNPs of *TaNAC26-D3.2* formed two haplotypes, Hap1 and Hap2, and germplasms with Hap2 exhibited better salt tolerance. Snp3, in exon 3 of *TaNAC26-D3.2,* causing a synonymous mutation, was developed into a Kompetitive Allele-Specific PCR marker, K3, to distinguish the two haplotypes, which can be further used for wheat germplasm screening or marker-assisted breeding. This study provides new genes and molecular markers for improvement of salt tolerance in wheat.

## 1. Introduction

Soil salinization, impacting nearly 20% of agricultural areas worldwide, poses a critical threat to global food production, with its severity exacerbated by climate change and population growth [1]. Serving as a staple food for most of the global population and an important source of carbohydrates and calories, common wheat (*Triticum aestivum* L., AABBDD) is susceptible to salt stress, which can hamper its growth by inducing osmotic stress and ion toxicity and ultimately lead to diminished yields or even plant death [2,3].

Transcription factors constitute approximately 7% of the coding regions in plant transcriptomes [4] and are pivotal in regulating gene expression, signal transduction, and stress-responsive functions, thus positioning them as promising candidates for genetic engineering of stress tolerance [5]. To date, more than 300,000 transcription factors have been reported in plants [6], of which the NAC (NAM-ATAF-CUC) family stands out as one of the largest transcription factor families. Typical NAC proteins consist of a well-conserved N-terminal DNA binding domain, which includes five subdomains (A–E), and a highly variable C-terminal transcriptional regulatory region [7,8]. NAC proteins are believed to be important regulators of plant functions in various aspects, such as morphogenesis, senescence, plant hormone homeostasis, and stress responses [6,9,10].

So far, *NAC* genes involved in salt tolerance have been identified and studied in various species. In *Arabidopsis*, *VNI2*, which was induced by high salinity, was reported as a positive regulator of resistance to salt stress [11]. Plants overexpressing *JUB1* exhibited a salt-tolerant phenotype, while *jub1* knockout mutants were hyper-sensitive to salt stress [12]. On the contrary, some salt stress-related *NACs* play a negative role in salt tolerance. *Arabidopsis* plants overexpressing *ANAC069* displayed increased sensitivity to salt stress by inhibiting ROS scavenging capability and proline biosynthesis, while *ANAC069* knockdown plants showed enhanced tolerance [13]. *NTL8*, induced by high salt, negatively regulates the salt response during germination [14]. *ANAC092*/*AtNAC2*/*ORE1* also negatively regulates salt tolerance [15,16]. Interestingly, overexpression of *ATAF1* in *Arabidopsis* leads to increased sensitivity to NaCl, while its ectopic expression in rice exhibits enhanced salt tolerance [17,18]. *NAC* genes are also well characterized in monocots. In rice, multiple *NAC* transcription factors positively regulate salt stress responses. *SNAC1* enhances tolerance to multiple abiotic stresses when introduced into rice and wheat [19,20]. Overexpression of *ONAC045* improves salt and drought tolerance [21]. *OsNAC5* and *OsNAC6*, induced by ABA, drought, and salt, enhance salt and drought tolerance when overexpressed [22,23]. *OsNAP*, *ONAC106*, and *ONAC022* positively regulate salt stress responses, though specific mechanisms vary [24,25,26]. *OsNAC5* acts as a transcriptional activator in abiotic stress responses without affecting rice growth [22]. Collectively, these *NACs* mediate stress tolerance through transcriptional regulation, though trade-offs between stress resistance and agronomic traits exist for certain family members like *OsNAC6* [23].

In wheat, several NAC transcription factors have been identified to mediate salt stress tolerance. *TaNAC2* enhances drought, salt, and freezing tolerance in *Arabidopsis* by upregulating stress-responsive genes and physiological indices [27]. *TaNAC29*, induced by various abiotic stresses, confers salt and drought tolerance during both vegetative and reproductive stages through the ABA signaling pathway and antioxidant enzyme systems [28]. Similar functions were reported for the other *TaNAC29*, a 306-amino acid protein-encoding gene that enhances salt tolerance via stress-related gene induction and activation of ROS-scavenging antioxidant enzymes [29]. Notably, while *TaNAC29* exhibits consistent stress resistance across growth stages, other *NACs* like *TaNAC2D* display stage-specific responses [30]. For example, *TaNAC2D*-overexpressing *Arabidopsis* showed increased sensitivity to salt and drought at the mature stage but improved tolerance at the seedling stage, suggesting that NAC functions may vary with developmental context and environmental conditions. *TaNAC47* [31] and *TaNAC67* [32], which are induced by drought, salt, cold, and ABA treatments, improve transgenic *Arabidopsis* tolerance to these stresses via upregulation of stress-responsive genes. Notably, overexpression of *TaNAC67* enhances physiological parameters, including cell membrane stability, chlorophyll retention, Na^+^ efflux, photosynthetic capacity, and water retention.

While several NAC transcription factors have been identified in wheat, the large size of the wheat NAC family leaves uncertainty about whether other members participate in salt stress responses. Here, we conducted a genome-wide investigation of the expression patterns of the *TaNAC* family under salt stress. Through transcriptome sequencing and association analysis, we identified a salt-responsive gene, *TaNAC26-D3.2*, and developed a diagnostic marker for its salt-tolerant haplotype, providing a valuable tool for molecular breeding applications.

## 2. Results

### 2.1. Isolation and Systematic Numbering of the TaNAC Gene Family

**Figure 1:**
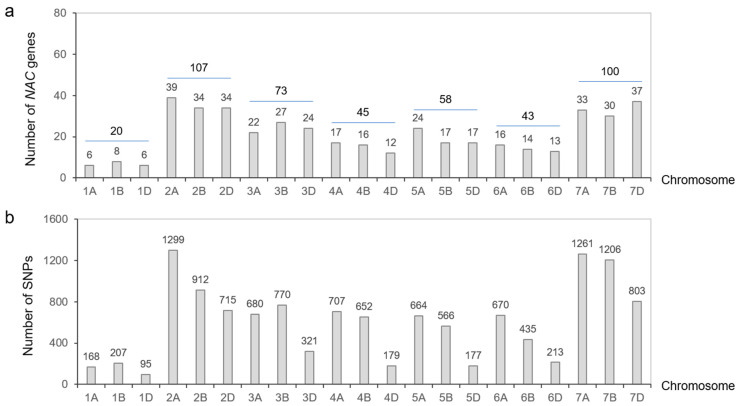
Chromosome distribution of *TaNAC* genes and their SNPs. (**a**) Number of *TaNAC* genes distributed on wheat chromosomes. (**b**) Number of SNPs derived from *TaNACs* on each chromosome.

A total of 446 sequences with the no apical meristem (NAM) domain were retrieved from the whole-protein sequences of common wheat ‘Chinese Spring’ and were distributed across all 21 chromosomes of wheat (Figure 1a). The homoeologous group 2 contained the highest number of *NACs* (107), while the homoeologous group 1 contained the fewest, with only 20.

**Figure 2:**
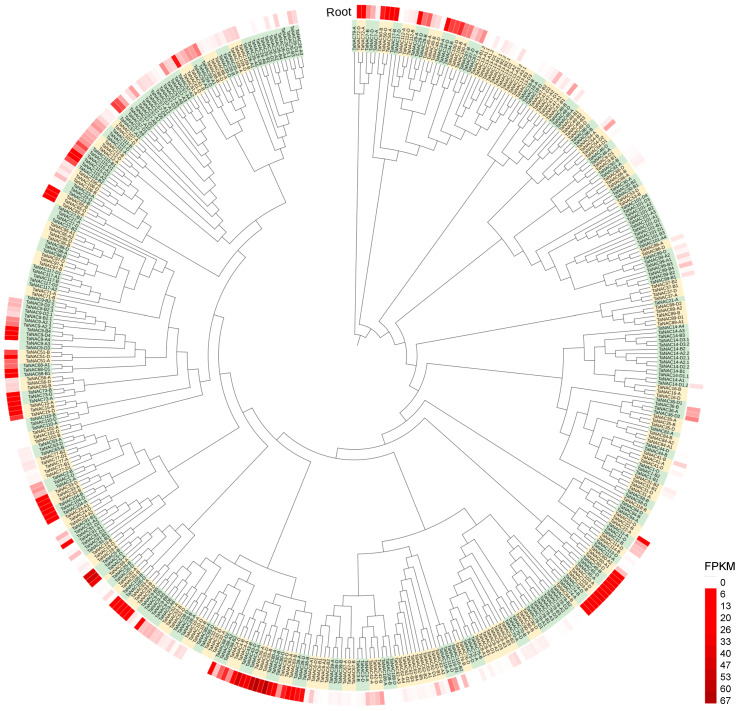
Phylogenetic tree and transcriptional level of *NAC* genes in wheat root. The tree was constructed by 466 wheat NAC protein sequences, and the different members were distinguished by green and yellow. These NACs were classified into 118 members based on subgenome homology and sequentially numbered from TaNAC1 to TaNAC118 according to their physical position on chromosomes 1~7. For each *TaNAC*, the FPKM value was obtained from the root RNA-seq data of wheat line CH7034 seedling and visualized in heatmap, with darker red indicating higher values.

These *NACs* were classified into 118 members based on subgenome homology and sequentially numbered from *TaNAC1* to *TaNAC118* according to their physical position on chromosomes 1~7 (Figure 2, Table S1). Among them, 112 members had two or three copies in the A, B, and D subgenomes, and the remaining six members each had a single copy. In addition, 32 *TaNAC* members (27.12%) underwent tandem duplication events and were found in gene clusters in the genome, with each gene cluster containing 2~9 tandem duplicated genes (Figure 2).

The copies of each *TaNAC* member in subgenomes did not invariably exhibit the same expression level. In the roots of wheat seedlings, on the whole, 145 *TaNAC* genes (32.51%) were expressed normally, 182 *TaNACs* (40.81%) showed the low transcript level with the FPKM value less than 1, and the remaining 119 *TaNACs* (26.68%) detected no expression (Figure 2, Table S1).

### 2.2. Transcriptional Response to Salt Stress of TaNACs

**Figure 3:**
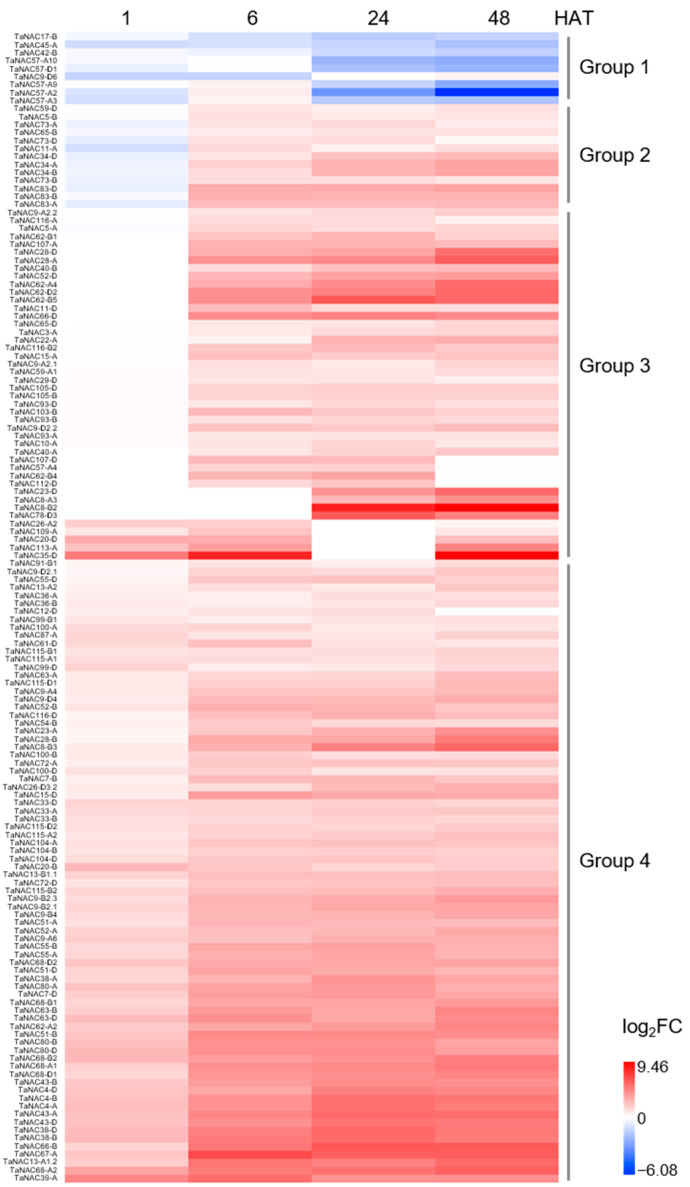
Differentially expressed *TaNAC* genes in root after NaCl stress at 1, 6, 24, and 48 h, compared with 0 h. Change in transcriptional level after treatment was defined as log_2_|FPKM_treatment_/FPKM_control (0 HAT)_| > 1. The 144 *TaNACs* differentially expressed at least at two treating time points were selected for display, with upregulation changes appearing in red and downregulation changes appearing in blue. These *TaNACs* were divided into four groups. HAT: hours after treatment.

Transcriptome sequencing of roots in CH7034 revealed that among the 446 *TaNACs*, 144 genes showed significant changes in expression levels at least two time points after salt stress (Table S2). These differentially expressed genes were divided into four groups (Figure 3). Group 1 exhibited a downregulation trend under salt stress, Group 2 downregulated its expression within 1 h after treatment (HAT) and then continued to increase thereafter, Group 3 showed specific upregulation of expression at two or three time points, and the remaining Group 4, containing the largest number of 78 genes, exhibited a successive upregulation trend after salt treatment (Figure 3).

### 2.3. Association Analysis Between TaNACs and Root Salt-Tolerance Phenotypes

**Figure 4:**
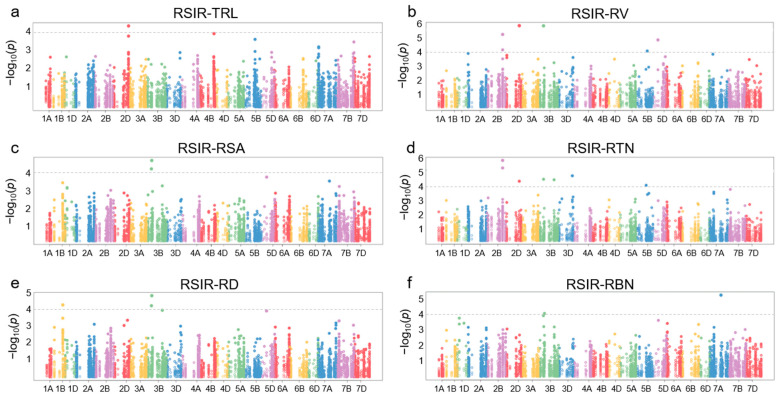
Manhattan plot of association analysis between *TaNAC* genes and root salt-tolerance phenotypes in 114 wheat accessions. (**a**) Relative salt-injury rate (RSIR) for total root length (TRL). (**b**) RSIR for root volume (RV). (**c**) RSIR for root surface area (RSA). (**d**) RSIR for root tip number (RTN). (**e**) RSIR for root diameter (RD). (**f**) RSIR for root branching number (RBN). The threshold is set to −log_10_*p* > 4 (i.e., *p* < 0.0001).

The sequences of *TaNACs* were BLAST (version 2.16.0) against the WheatUnion database, and a total of 12,700 single nucleotide polymorphisms (SNPs) were identified across all 21 chromosomes of wheat in 114 wheat germplasms (Figure 1b, Table S3). For the wheat germplasms, six salt-tolerant phenotypes associated with roots, including total root length (TRL), root tip number (RTN), root surface area (RSA), root diameter (RD), root volume (RV), and root branching number (RBN), were identified, and the relative salt-injury rates (RSIR) were calculated based on data from the control group and NaCl treatment group. Association analysis results showed that 20 SNPs distributed on chromosomes 1B, 2B, 2D, 3B, 3D, 5B, 5D, and 7A were correlated with root phenotypes involving salt tolerance (*p* < 0.0001) (Figure 4, Table S4).

After further screening, six high-confidence SNPs with a minimum allele frequency > 10% and a maximum missing rate of <5% were selected, including 1430[A/T], 0779[A/G], 2830[A/G], 3581[G/A], 4718[G/T], and 5088[T/C] (Table 1). Among them, 1430[A/T] from *TaNAC3-B* was associated with RSIR-RD, 0779[A/G] from *TaNAC26-D3.2* was associated with RSIR-TRL and RSIR-RV, 2830[A/G] from *TaNAC31-B1* was associated with RSIR-RSA and RSIR-RD, 3581[G/A] from *TaNAC32-B* was associated with RSIR-RV, RSIR-RSA, RSIR-RTN, and RSIR-RD, 4718[G/T] from *TaNAC33-B* was associated with RSIR-RBN, and 5088[T/C] from *TaNAC40-B* was associated with RSIR-RTN.

### 2.4. Candidate TaNAC Genes Responding to Salt Stress in Roots

**Figure 5:**
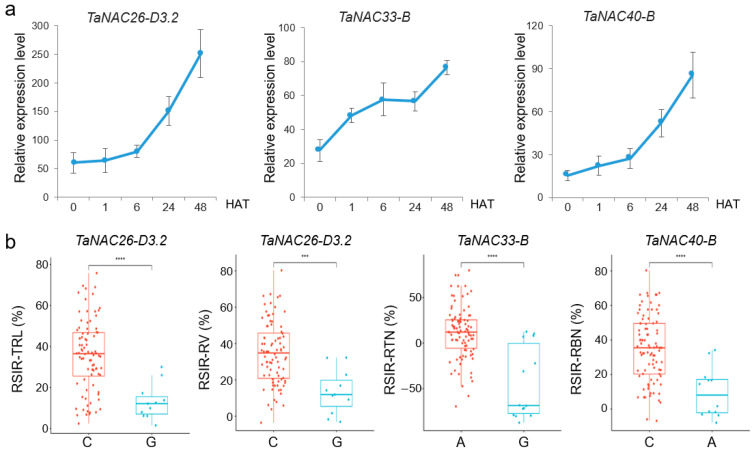
Three candidate *TaNAC* genes responding to salt stress. (**a**) Relative expression level of three *TaNACs* after NaCl treatment. The bars indicate the standard error. HAT: hours after treatment. (**b**) Significant differences analysis in root phenotypes corresponding to genotypes of high-confidence SNPs from the candidates. RSIR: relative salt-injury rate; TRL: total root length; RV: root volume; RSA: root surface area; RTN: root tip number; RD: root diameter; RBN: root branching number. *** indicates *p* < 0.001, and **** indicates *p* < 0.0001, according to the *t*-test.

According to the RNA-seq results, we selected three *NAC* genes that were continuously upregulated after salt stress, including *TaNAC26-D3.2*, *TaNAC33-B*, and *TaNAC40-B*, and the further RT-qPCR experiments have shown consistent results (Figure 5a). Furthermore, these three genes were associated with root salt tolerance phenotypes based on association analysis (Figure 5b, Table S5). SNP 0779[A/G], located in exon 3 of *TaNAC26-D3.2*, caused a synonymous mutation and involved RSIR-TRL and RSIR-RV; SNP 4718[G/T] was located in intron 3 of *TaNAC33-B* and was related to RSIR-RTN; and SNP 5088[T/C], located in intron 4 of *TaNAC40-B*, was correlated with RSIR-RBN.

### 2.5. Functional Verification of TaNAC26-D3.2

**Figure 6:**
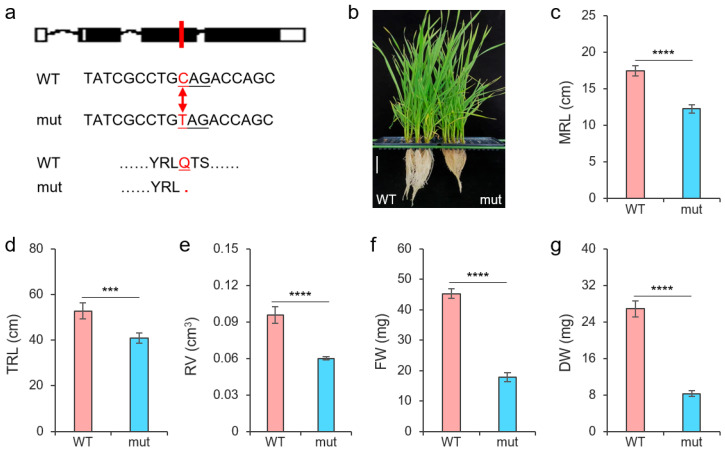
Effect of *TaNAC26-D3.2* on salt tolerance in wheat roots. (**a**) An EMS-mediated C to T mutation resulting in a premature stop codon in exon 2 of *TraesCS2D02G576400* in the *tanac26-d3.2* mutant. (**b**) Appearance of roots of wide type (WT) and *tanac26-d3.2* mutant. Scale bar, 5 cm. (**c**–**g**) Phenotypes of WT and *tanac26-d3.2* mutant. MRL: maximum root length; TRL: total root length; RV: root volume; FW: root fresh weight; DW: root dry weight. *** indicates *p* < 0.001, and **** indicates *p* < 0.0001, according to the *t*-test.

To verify the effect of *TaNAC26-D3.2* on salt tolerance in wheat, we identified a mutant line with a C to T mutation in its exon 2, which produced a premature stop codon and led to a truncated protein (Figure 6a), to observe changes in roots after salt stress. As expected, we observed the decreased root length of the *tanac26-d3.2* mutant compared to the wild type after treatment (Figure 6b). Both the maximum root length (MRL) and the TRL of the mutant were shorter than that of the wild type (Figure 6c,d). Moreover, compared with the wild type, the *tanac26-d3.2* mutant roots showed less volume and reduced biomass after being subjected to salt stress (Figure 6e–g). This result suggested that *TaNAC26-D3.2* positively regulated the salt tolerance of plants.

### 2.6. Haplotypes of TaNAC26-D3.2

**Figure 7:**
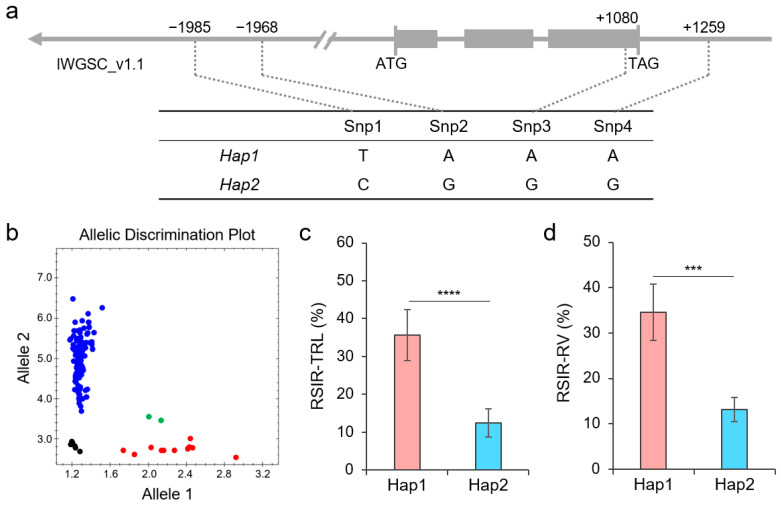
Haplotypes of *TaNAC26-D3.2*. (**a**) Location of four SNPs in the gene. The gray box indicates an exon. (**b**) Genotyping result of KASP marker K3 in 114 wheat germplasms. (**c**,**d**) Phenotypic differences in RSIR-TRL and RSIR-RV between Hap1 and Hap2. *** indicates *p* < 0.001, and **** indicates *p* < 0.0001, according to the *t*-test.

Based on the WheatUnion database, we found four highly confident SNPs, including the 0779[A/G] mentioned earlier, from the *TaNAC26-D3.2* gene region covering 2000 bp before the start codon, 500 bp after the stop codon, and the complete gene sequence between the start codon and the stop codon. Among them, Snp1 and Snp2 are located at −1985 bp and −1968 bp in the promoter region, respectively; Snp3 is the 0779[A/G] that caused a synonymous mutation, while Snp4 is located at position 1259 bp in the 3′-UTR of *TaNAC26-D3.2* (Figure 7a). These four SNPs from *TaNAC26-D3.2* displayed two haplotypes, Hap1 and Hap2, in the 114 wheat germplasms used in this study. We transformed Snp3 into a KASP marker, K3, which accurately distinguished the two haplotypes of *TaNAC26-D3.2*, showing an excellent genotyping efficiency (Figure 7b). Association analysis showed that K3 was significantly correlated with the RSIR-TRL and the RSIR-RV (Figure 6c,d). Hap2 had lower RSIR values than Hap1, indicating enhanced salt tolerance.

## 3. Discussion

### 3.1. Systematic Numbering of Wheat NAC Family

The NAC family is one of the largest transcription factor families in plants. Multiple *NAC* genes have been reported in wheat, involving organ development, disease resistance, stress tolerance, and so on [9,10]. However, the nomenclature of NAC varied among studies, and the inconsistent naming standards have caused confusion for researchers. For example, the salt tolerance gene *TaNAC29* reported by Huang et al. (KT783450) [28] and that reported by Xu et al. (KP657687) [29] exhibited a low sequence similarity. For the sake of consistency and convenience in research, this study isolated all the *NAC* genes in wheat and divided them into 118 members, which were named as *TaNAC1*~*TaNAC118* based on their phylogenetic analysis results and genomic location information. Then we performed sequence alignment for the reported wheat *NAC* genes and our *TaNAC* members, in which the E value of 0 and the sequence similarity of 100% were used to identify *NACs* for nomenclature revision, which can help researchers better understand the research progress of NAC.

For instance, as shown in Table 2, *TaNAC29* (KT783450) [28] and *TaNAC29* (KP657687) [29], mentioned before, were two completely different genes, and they correspond to *TaNAC9-A6* and *TaNAC68-D2* in this study, respectively. We also found that some reported *TaNACs* are actually the same gene. *TaRNAC1* [33], *TaNAC14* [34], and *TaNAC100* [35] are *TaNAC15*; *TaNAC1* [36] and *TaNAC-S* [37] are *TaNAC106*; *TaNAC5D-2* [38] and *TaNACL-D1* [39] are *TaNAC66-D*; *NAM-A1* [40] and *TaSNAC8-6A* [41] are *TaNAC85-A*; and *TaNAC69* [42] and *TaNAC29* [29] are two copies of *TaNAC68* in the B- and D-subgenomes, respectively; similarly, *TaNAC2-5A* [43], *TaNAC2* [27], *TaNAC2a* [44], and *TaNAC2D* [30] are subgenome copies of *TaNAC80*; and *TaNAC47* [31] and *TaNAC67* [32] are subgenome copies of *TaNAC83*. These *TaNAC* genes with pleiotropy regulate multiple phenotypes, which has great potential for application in breeding.

Moreover, the new names of *TaNAC* genes reflect genomic translocations (e.g., the A subgenome copies of *TaNAC61~63* were located in the 4AL/5AL translocation region of the wheat genome [45]), tandem duplication, and their subgenomic homologous relationships, which is beneficial for revealing the evolutionary process, functional redundancy, and expression patterns of the family.

### 3.2. Response of TaNAC Genes to Salt Stress in Wheat Roots

Roots play a crucial role in salt response, since they are the first organs that directly sense and cope with osmotic stress and toxic conditions in saline soils [46]. In the model plant Arabidopsis, NAC transcription factors have been reported to participate in root responses to salt stress by functioning in various processes, such as osmotic adjustment and stress signaling pathways [47]. Within the 446 *TaNAC* genes isolated in this study, 144 genes were differentially expressed in roots at least two time points after NaCl treatment. Among them, *TaNAC9-A6* (previously reported as *TaNAC29*), *TaNAC15* (previously reported as *TaNAC14*), *TaNAC68-B2* (previously reported as *TaNAC69-1*), *TaNAC68-D2* (previously reported as *TaNAC29*), *TaNAC72-D* (previously reported as *SIP1*), *TaNAC80-A* (previously reported as *TaNAC2*), *TaNAC80-D* (previously reported as *TaNAC2D*), *TaNAC83-A* (previously reported as *TaNAC47*), and *TaNAC83-B* (previously reported as *TaNAC67*) have been confirmed to regulate salt tolerance in wheat [27,28,29,30,31,32,34,48,49], and the remaining NACs were reported for the first time in response to salt stress.

Our study also revealed the response of tandem duplicated NAC genes to salt stress. In total, 32 *TaNAC* members experienced tandem duplication events, 12 of which had duplicated genes for each homologous copy. In general, clustered genes, with their overlapping or unique regulatory functions and mutual regulation capabilities, are particularly advantageous for regulating pathways involved in defense [47].

For example, in the wheat salt tolerance site *TaCYP81D* containing five tandem duplicate genes, mutation in one gene led to a significant increase in the expression levels of other genes, maintaining the plant’s tolerance to salt stress [50]. Among the 12 *TaNAC* members with large gene clusters mentioned earlier, all tandem duplicate genes of *TaNAC115* maintained high expression levels after NaCl treatment, and tandem duplicate genes of eight members, including *TaNAC8*, *TaNAC9*, *TaNAC13*, *TaNAC26*, *TaNAC47*, *TaNAC57*, *TaNAC62*, and *TaNAC91*, partially responded to salt stress. These *TaNAC* clusters may enhance wheat’s tolerance to adverse natural variations in the genome, thereby maintaining its salt tolerance. However, there are three members, *TaNAC14*, *TaNAC78*, and *TaNAC101*, whose tandem duplicate genes were either not expressed or showed low expression levels, reflecting the redundancy of gene function.

### 3.3. TaNAC26-D3.2 Positively Regulates Salt Tolerance

In this study, three candidate *NAC* genes, including *TaNAC26-D3.2*, *TaNAC33-B*, and *TaNAC40-B*, were identified based on the results of RNA-seq and association analysis. Using an EMS-mutant line, we confirmed that knocking out *TaNAC26-D3.2* led to a decrease in root tolerance to salt stress, indicating that *TaNAC26-D3.2* positively regulated plant salt tolerance. However, the relevant molecular mechanism of salt tolerance still needs further investigation. Salt stress reshapes root system architecture by differentially affecting the growth rate of the main root and lateral roots through hormone-mediated pathways [51]. Multiple interaction models have been identified in Arabidopsis, such as the ANAC092/AtNAC2/ORE1 model involving ethylene and auxin signaling pathways, regulating lateral root development [15,16], as well as the recently reported SMB-AUX1-auxin signaling module regulating root halo tropism [52]. Therefore, identification of interaction factors from hormone signaling pathways for *TaNAC26-D3.2* will be conducted in our future studies. Moreover, Chi et al. [34] revealed that *tae-miR164* inhibits root development and reduces salinity tolerance by down-regulating the expression of *TaNAC14* (*TaNAC15* in this study). We will identify whether *TaNAC26-D3.2* is regulated by miRNA.

In addition, *TaNAC26-D3.2* has also been proven to negatively regulate plant resistance to stripe rust [53]. Silencing of *TaNAC30* (*TaNAC26-D3.2* in this study) by virus-induced gene silencing inhibited colonization of the virulent isolate of *Puccinia striiformis* f. sp. *tritici* by inducement of the accumulation of H_2_O_2_ [53]. Accordingly, the impact of *TaNAC26-D3.2* on plant disease resistance should be considered when applying it to salt tolerance improvement in breeding. One KASP marker, K3, was developed on Snp3 from exon 3 of *TaNAC26-D3.2*, which could be further used for wheat germplasm screening or marker-assisted selection.

## 4. Materials and Methods

### 4.1. Plant Materials

A salt-tolerant wheat breeding line, CH7034 [54], which was developed by the College of Agronomy of Shanxi Agricultural University and with a pedigree of Jing411/Xiaoyan7430//Zhong8601, was used for RNA sequencing, and a set of wheat germplasm containing 114 accessions [55] was used for association analysis. Mutant lines obtained by ethylmethanesulfonate mutagenesis and their wild-type wheat cv. Kenong 9204 were used to verify the phenotype of *TaNAC26-D3.2*.

### 4.2. Isolation of NAC Sequences from Wheat Genome

Isolation of the gene family was performed referring to our previous method [56]. In brief, hidden Markov model files of the NAC superfamily (PF02365, http://pfam.xfam.org/, accessed on 24 November 2023) were employed to retrieve annotated protein sequences of Chinese Spring (version 1.1, http://wheat-urgi.versailles.inra.fr/, accessed on 10 March 2023) in the HMMER software (version 3.0). The obtained sequences were confirmed on the SMART database (http://smart.embl-heidelberg.de/, accessed on 31 December 2023) for containing the NAM domain. The number of members was determined based on the homoeologue of A/B/D subgenomes (https://plants.ensembl.org/Triticum_aestivum/, accessed on 31 December 2023), and genes within 10 Mb were defined as tandem duplicated genes and numbered according to their linear arrangement on the chromosome. The phylogenetic tree was constructed by TaNAC protein sequences using the neighbor-joining method in MEGA (version 6.0). Sequence alignment was performed for the reported wheat *NAC* genes and our *TaNAC* members, using the BLAST package (version 2.16.0) with the E value of 0 and the sequence similarity of 100%.

### 4.3. Transcriptional Data of TaNAC Genes After Salt Treatment

CH7034 seedlings were grown in half-strength Hoagland’s culture solution in a growth chamber under a 22/16 °C (day/night) temperature regime and a 16/8 h (light/dark) photoperiod with 60% relative humidity. When the seedlings grew to the three-leaf stage, they were exposed to 250 mmol/L NaCl for salt-stress treatment [54,55,56], and the roots were harvested at 0, 1, 6, 24, and 48 h, with three biological replicates for each sample. Total RNA was extracted by TRIzol reagent (Invitrogen, Carlsbad, CA, USA) according to the manufacturer’s instructions, and the cDNA libraries were constructed using the TruSeq RNA Sample Preparation Kit v2 (Illumina, San Diego, CA, USA). Transcriptome sequencing was performed on the Illumina platform (Biomarker Tech., Beijing, China). Clean reads were obtained by removing low-quality reads containing adapters and poly-N (>10%) or with a quality score < 30 from the raw data and mapping them to the Chinese Spring reference genome (RefSeq v1.0, http://wheat-urgi.versailles.inra.fr/, accessed on 20 May 2024). The transcript level of each gene was measured with FPKM values calculated from the following formula: FPKM = cDNA Fragments/Mapped Fragments (Millions) * Transcript Length (kb), where “cDNA Fragments” represents the number of fragments mapped to a certain transcript, “Mapped Fragments (Millions)” represents the total number of fragments mapped to the transcript, measured in units of 10^6^, and “Transcript Length (kb)” represents the length of the certain transcript, measured in units of 10^3^ bases. The wheat NAC sequences obtained previously were then compared with these assembled genes to obtain their expression data at different time points under salt treatment, which were measured by fragments per kilobase of exon model per million mapped fragments (FPKM) values. Differential expression analysis between the control group (0 h of NaCl treatment) and the salt-stress group (1, 6, 24, and 48 h of NaCl treatment) was performed using the DESeq package (version 2), with a threshold of |log_2_FoldChange| ≥ 1 and FDR ≤ 0.01.

### 4.4. RT-qPCR

Three-leaf-stage seedlings were subjected to salt stress with 250 mmol/L NaCl, and roots were collected at 0, 1, 6, 24, and 48 h after treatment. Total RNA was extracted using an RNA extraction kit (Tianmo Bio, Beijing, China) and reverse-transcribed into cDNA using a reverse transcription kit (Takara Bio, Shiga, Japan). RT-qPCR was performed on the QuantStudio 3 Real-time PCR System (Applied Biosystems, Carlsbad, CA, USA), using Premix Ex Taq II enzyme (Takara Bio, Shiga, Japan) and primer pairs for *NAC26-D3.2* (qF: 5′-CAGGGACAAGCCCGTGACA-3′; qR: 5′-GCGTGCTCGCTGGTCTGC-3′), *NAC33-B* (qF: 5′-CTGGTTGCAGTACTAATGGAT-3′; qR: 5′-CCTTAGGATGGAATCCAAATC-3′), *NAC40-B* (qF: 5′-GTTTCCTGCCGGTGTCAAG-3′; qR: 5′-GCCATTTCTTGTCACACCTG-3′), and the internal reference gene *ACTIN* (F: 5′-GGAACTGGCATGGTCAAGGCTG-3′; R: 5′-CCCATCCCCACCATCACACC-3′). Each reaction was repeated three times, and the results were analyzed using the 2^−Δ*CT*^ and fold-change method, wherein the Δ*CT* was calculated using the following formula: Δ*CT* = *CT* (target gene) − *CT* (reference gene).

### 4.5. Association Analysis of TaNAC Genes

RSIR-R was investigated for 114 wheat germplasm resources after treatment with 250 mmol/L NaCl for 10 days since the three-leaf-stage seedling stage, which was under the same growth condition as the CH7034 line. A root scanner (MicroTek, Shanghai, China) was calibrated and used to scan the wheat roots after 7 days of H_2_O treatment (CK) and NaCl treatment to obtain the total root length (TRL), root surface area (RSA), root volume (RV), root diameter (RD), root tip number (RTN), and root branching number (RBN). Ten individual plants per germplasm were tested, and the average of their values was used for subsequent analysis. The relative salt-injury rate of each root phenotype for wheat accession was calculated using the following formula: RSIR-R (%) = (X_CK_ − X_NaCl_)/X_CK_ × 100%. The experiment was repeated three times, and the average value was taken. The TaNAC family sequences were submitted to the WheatUnion database (http://wheat.cau.edu.cn/WheatUnion/, accessed on 7 July 2024), and SNPs in the coding regions and 2000 bp upstream from the start codon of the genes were retrieved and analyzed for association with the root salt injury index in the 114 wheat germplasms using the GAPIT program (version 3) in R (version 4.3.1). The significance threshold was set at −log_10_*p* > 4 (i.e., *p* < 0.0001).

### 4.6. Association Analysis of TaNAC16-B

According to our previous method [57], a KASP marker was developed on a SNP from exon 3 of *TaNAC26-D3.2* (K3-F1: 5′-GAAGGTGACCAAGTTCATGCTggctgtccaggaagttccaA-3′; K3-F2: 5′-GAAGGTCGGAGTCAACGGATTggctgtccaggaagttccaG-3′; K3-R: 5′-CGCGGGCTATGATGTCGTG-3′) and used for genotyping the germplasm. The association between different genotypes of K3 and the relative salt-injury rate of each root phenotype was analyzed according to the *t*-test, setting the significant threshold at *p* < 0.01. The reaction system (5 μL) contained 2.5 μL of KASP Master Mix (LGC Biosearch Technologies, Petaluma, CA, USA), 0.07 μL of primer mixture, and 2.43 μL of DNA solution (30 ng/μL). The KASP reactions were conducted using the QuantStudio 3 Real-Time PCR System mentioned above with the following program: denaturation at 94 °C for 10 min, ten cycles of touchdown PCR (94 °C for 20 s; touchdown at 60 °C initially and decreasing by −0.6 °C per cycle for 60 s), and 40 additional cycles (94 °C for 20 s; 55 °C for 60 s). PCR products were detected in a fluorescence scanner under FAM and HEX channels.

### 4.7. Investigate for EMS-Mutant

The three-leaf-stage seedlings of the *tanac26-d3.2* mutant and its wild-type cv. Kenong9204 were exposed to 150 mM NaCl for 7 days. Their root phenotypes were measured by the method mentioned in Section 4.4. Then, the roots of the mutant and wild-type plants were cut off and weighed using an electronic balance to obtain fresh weight (FW). The cut roots were then dried in an oven at 42 °C for 2 days to measure dry weight (DW). The mutation site of the mutant was confirmed according to our previous method [58]. Briefly, one pair of specific primers, mF (5′-GTACTTCTTTAGCTTCAAGGAT-3′) and mR (5′-GGCCTCTGGTTCGGGGTT-3′), was used for amplifying a segment containing exon 2 of *TaNAC26-D3.2* with the annealing temperature of 58 °C. The PCR products were recovered after agarose gel electrophoresis and then sequenced. The sequences of *TaNAC26-D3.2*, between the wild-type and the mutant, were compared using SeqMan software (DNASTAR Inc., Madison, WI, USA) to confirm the mutational site.

## 5. Conclusions

In this study, 118 members containing 446 genes of the *TaNAC* family were isolated from the whole genome of common wheat. One hundred forty-four *TaNAC* genes were differentially expressed at least two time points after NaCl treatment in the roots of salt-tolerant line CH7034. Association analysis was conducted on 114 wheat germplasms, and six high-confidence SNPs from six *TaNAC* genes, including *TaNAC26-D3.2*, were significantly correlated with root salt-tolerance phenotypes. The *tanac26-d3.2* mutant roots showed decreased length, volume, and biomass after salt stress. *TaNAC26-D3.2* exhibited two haplotypes in the tested germplasms, with Hap2-typed germplasms corresponding to better salt tolerance than the Hap1-typed germplasms. A KASP marker was developed on a SNP detected in intron 3 of *TaNAC26-D3.2*, which can be further used for wheat germplasm screening or marker-assisted breeding.

## Figures and Tables

**Table 1:** Highly confident SNPs that significantly correlated with root salt-tolerance phenotypes.

Scheme 1.	Chromosome	Position (IWGSC v1.0)	*TaNAC* Genes	MAF	RSIR	*q* Value
1430[A/T]	1B	478581430	*TaNAC3-B*	0.26	RD	5.11 × 10^−5^
0779[A/G]	2D	639820779	*TaNAC26-D3.2*	0.11	TRL	4.42 × 10^−5^
					RV	1.67 × 10^−6^
2830[A/G]	3B	199702830	*TaNAC31-B1*	0.42	RSA	5.16 × 10^−5^
					RD	5.73 × 10^−5^
3581[G/A]	3B	214283581	*TaNAC32-B*	0.11	RV	1.71 × 10^−6^
					RSA	1.64 × 10^−5^
					RTN	1.91 × 10^−5^
					RD	1.45 × 10^−5^
4718[G/T]	3B	244384718	*TaNAC33-B*	0.16	RBN	9.14 × 10^−5^
5088[T/C]	3B	647245088	*TaNAC40-B*	0.11	RTN	2.09 × 10^−5^

**Table 2:** The correspondence between *TaNAC* genes in this study and reported *TaNACs*.

Gene Name in This Study	Reported Names in References
*TaNAC9-A6*	*TaNAC29* [28]
*TaNAC68-D2*	*TaNAC29* [29]
*TaNAC15*	*TaRNAC1* [33], *TaNAC14* [34], *TaNAC100* [35]
*TaNAC106*	*TaNAC1* [36], *TaNAC-S* [37]
*TaNAC66-D*	*TaNAC5D-2* [38], *TaNACL-D1* [39]
*TaNAC85-A*	*NAM-A1* [40], *TaSNAC8-6A* [41]
*TaNAC68*	*TaNAC69* [42], *TaNAC29* [29]
*TaNAC80*	*TaNAC2-5A* [43], *TaNAC2* [27], *TaNAC2a* [44], *TaNAC2D* [30]
*TaNAC83*	*TaNAC47* [31], *TaNAC67* [32]

## Data Availability

Data are contained within the article and Supplementary Materials.

## Supplementary Materials

The following supporting information can be downloaded at: https://www.mdpi.com/article/10.3390/plants14152318/s1, Table S1: Detailed information of *TaNAC* family members, Table S2: One hundred forty-four *TaNACs* differentially expressed at two treating time points, Table S3: One hundred fourteen wheat germplasms used in association analysis, Table S4: Twenty SNPs correlated with salt-tolerance phenotypes in roots, Table S5: FPKM values of three candidate *TaNAC* genes at 0, 1, 6, 24, and 48 HAT.
